# Supplementary figures and images for: Fatty acid metabolism in neutrophils promotes lung damage and bacterial replication during tuberculosis
Source: PLoS Pathog. 2024 Oct 4;20(10):e1012188. doi: 10.1371/journal.ppat.1012188 (PMC11482725; doi:10.1371/journal.ppat.1012188)

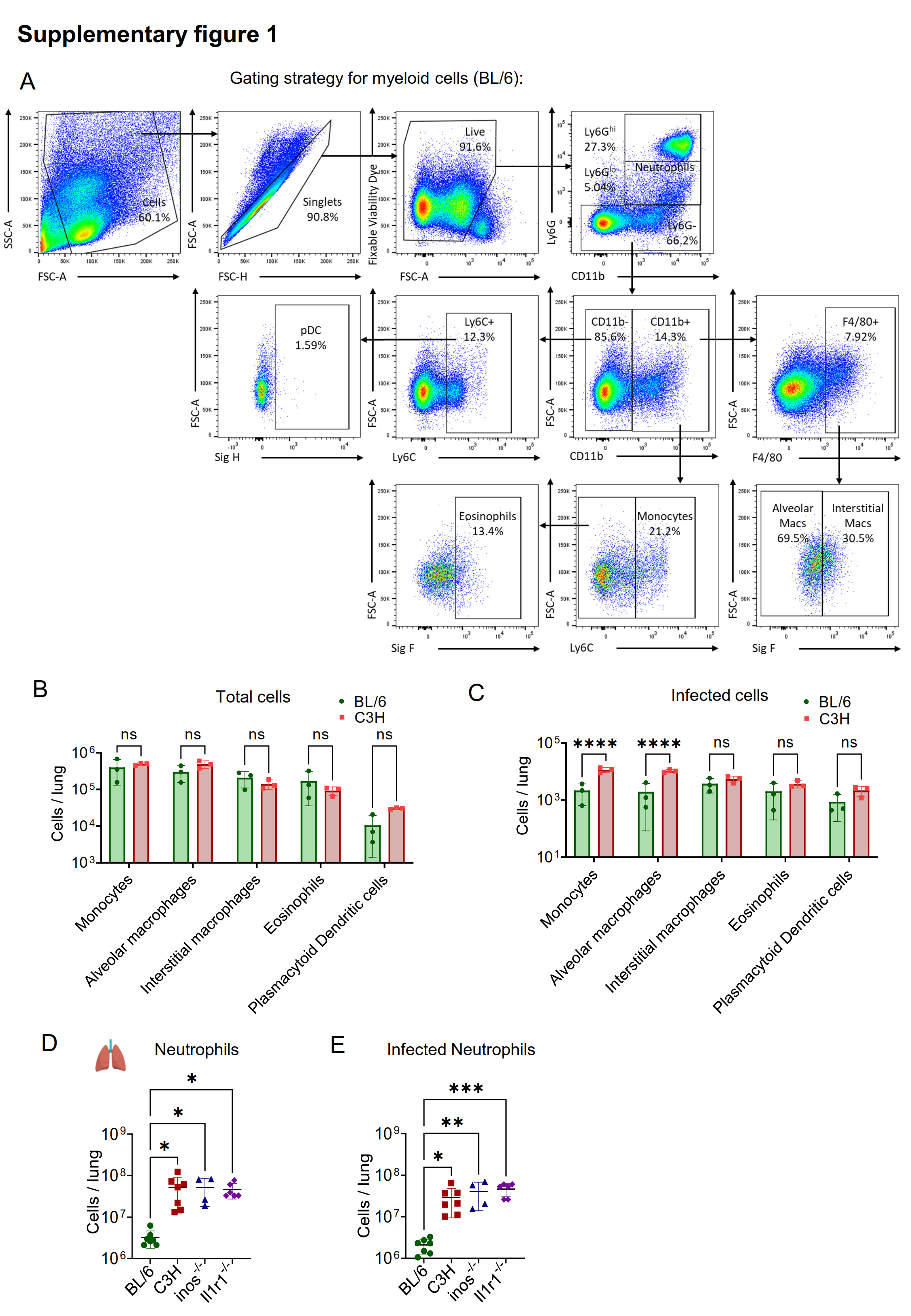

Supplement: S1 Fig — (A) The gating strategy used to identify myeloid cells from total lung single-cell suspensions in BL/6 mouse lungs, delineating Neutrophils: Live CD11b+ Ly6G+; Monocytes: Live CD11b+ Ly6G- Ly6C+; Alveolar Macrophages: Live CD11b+ Ly6G- F4/80+ Siglec F+; Interstitial Macrophages: Live CD11b+ Ly6G- F4/80+ Siglec F-; Eosinophils: Live CD11b+ Ly6G- Ly6C- Siglec F+; Plasmacytoid Dendritic cells (pDC): Live CD11b- Ly6G- Ly6C+ Siglec H+. (B) Enumeration of monocytes, alveolar macrophages, interstitial macrophages, eosinophils, and plasmacytoid dendritic cells in the lungs of resistant BL/6 and susceptible C3H mice at day 29 pi. (C) Counts of infected monocytes, alveolar macrophages, interstitial macrophages, eosinophils, and plasmacytoid dendritic cells in BL/6 and C3H mice at 29 dpi. (D) 6-8-week-old BL/6, C3H, inos-/-, and Il1r1-/- mice were infected with Mtb HN878 smyc’::mCherry SSB-GFP and evaluated at 29 dpi. Total live neutrophils (Live CD11b+ Ly6G+) numbers are shown for each mouse strain. Infected neutrophils (Live CD11b+ Ly6G+ smyc’::mCherry+) counts are presented for each mouse group. Sample sizes were n = 3–7 mice per group, representative of 2 experiments. Error bars represent Mean ± SD. Statistical analyses were performed using two-way ANOVA for (B, C) and ordinary one-way ANOVA for (D, E). Tukey’s multiple comparison tests were used to calculate statistical significance. Significance is indicated as *p<0.05, **p<0.01, ***p<0.001, ****p<0.0001; ns indicates non-significant differences. Clip art/Images within figure panels were created with www.BioRender.com. (TIF) [file ppat.1012188.s001.tif]

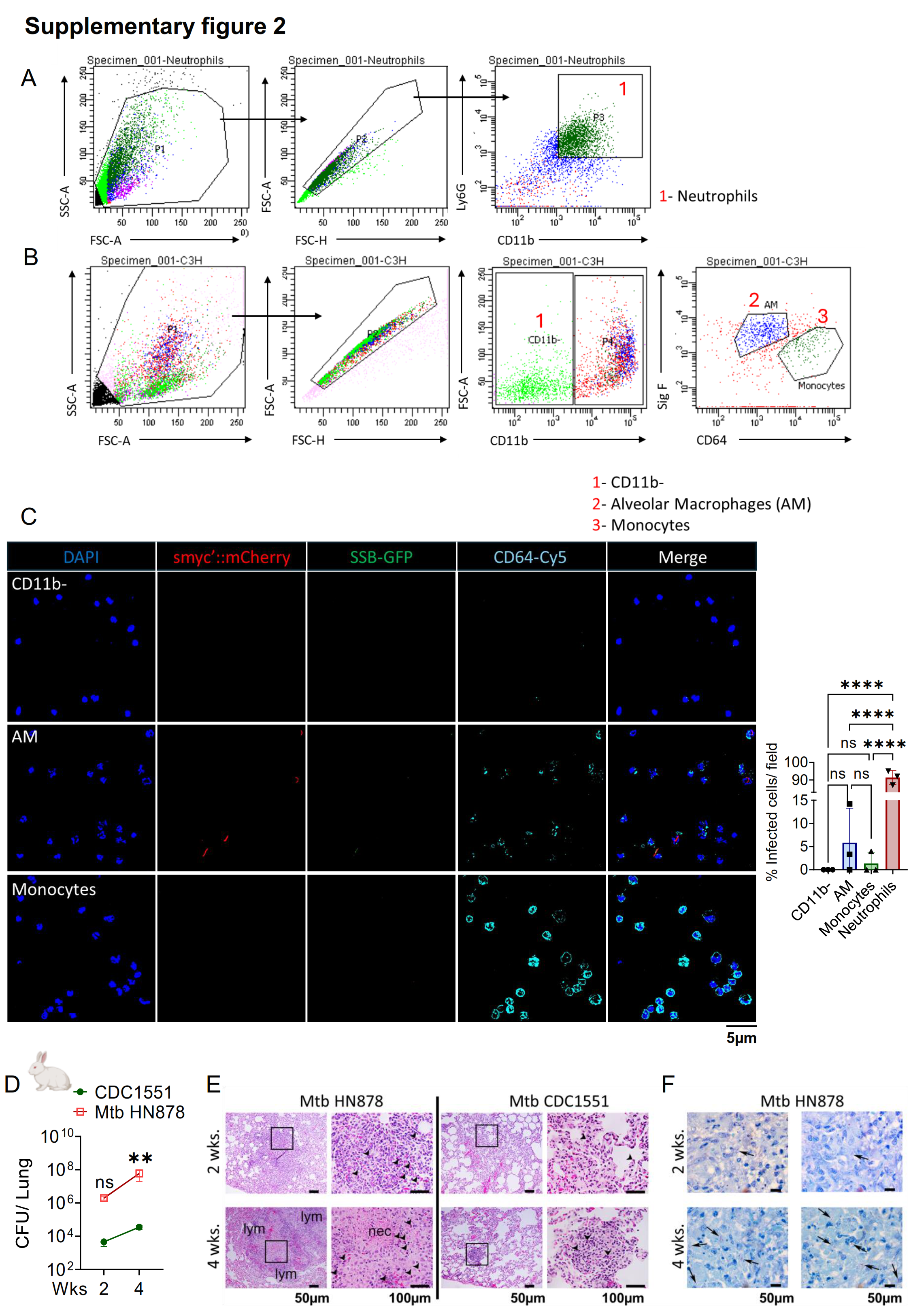

Supplement: S2 Fig — (A) Gating strategy for sorting neutrophils from C3H mouse lungs at 29 dpi: CD11b+ Ly6G+, prepared for confocal microscopy as shown in Fig 1F. (B) Description of the gating strategy for isolating CD11b- cells, alveolar macrophages (CD11b+ CD64lo, Siglec F+), and monocytes (CD11b+ CD64+, Siglec F-) from C3H mouse lungs at day 29 pi. (C) Representative confocal microscopy images of the sorted cell types after cytospin preparation. CD64 is visualized with Cy5, smyc’::mCherry+ identifies Mtb bacilli, and SSB-GFP foci are highlighted, with DAPI used as a counterstain. The top panel depicts CD11b- cells without CD64 or bacterial presence. The middle panel shows alveolar macrophages characterized by low CD64 levels and intracellular Mtb. The bottom panel displays monocytes with CD64 and absence of bacteria. Cell quantification from three mice is provided, indicating the percentage of cells harboring bacteria from CD11b-, AM, Monocytes and Neutrophils (Right). (D) Analysis of the bacterial burden in rabbit lungs infected with strains Mtb HN878 and CDC1551 at 2- and 4-weeks pi, using CFU counts. (E) Immunohistochemical evaluation of rabbit lung tissue infected with Mtb HN878 (left panel) and Mtb CDC1551 (right panel) at 2- and 4-weeks pi. Enlarged areas are indicated by square boxes in the left panels, with arrows in the right panels highlighting neutrophils, areas of necrosis, and lymphocytes. (F) Acid-fast staining of Mtb HN878 in infected rabbit lungs at 2- and 4-weeks pi, indicating bacterial presence and distribution. Sample sizes were n = 3 mice per group; 3 fields of view per mouse for (C) and n = 4 rabbits per group for (D). Error bars represent Mean ± SD. Statistical analyses were performed with ordinary one-way ANOVA for (C) and two-way ANOVA for (D). Tukey’s multiple comparison tests were applied to determine statistical significance. Significance levels are indicated: *p<0.05, **p<0.01, ***p<0.001, ****p<0.0001; ns denotes a non-significant result. Clip art/Image [file ppat.1012188.s002.tif]

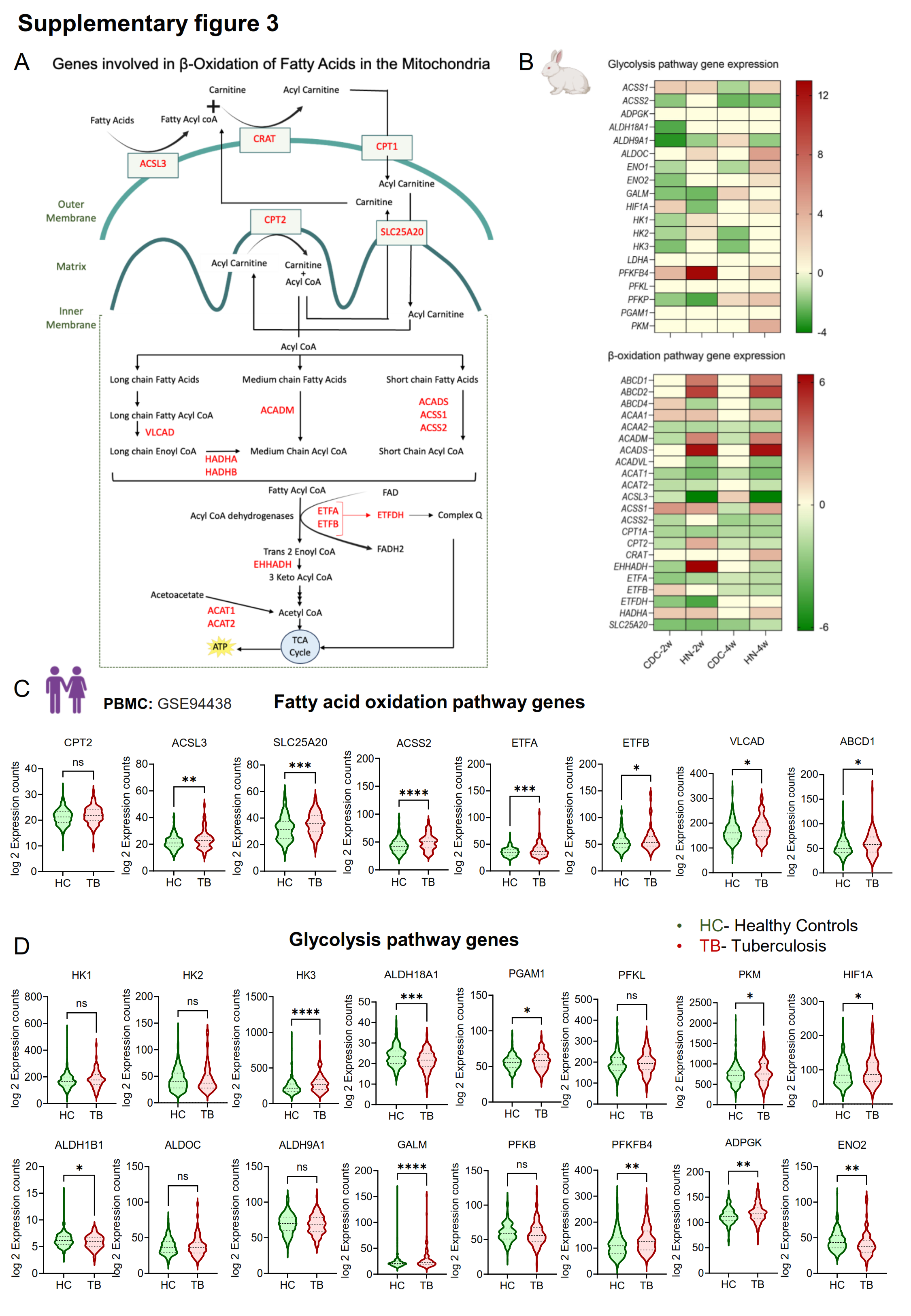

Supplement: S3 Fig — (A) Diagram illustrating genes associated with fatty acid metabolism. Highlighted in red are genes expressed in neutrophils from mice infected with Mtb HN878. (B) Heatmaps of Gene Expression: Differential gene expression related to glycolysis and β-oxidation pathways in rabbit lungs at 2- and 4-weeks post-infection with Mtb CDC1551 and Mtb HN878, as determined by genome-wide microarray analysis; (n = 4 rabbits/ group). (C) Fatty Acid Oxidation Gene Expression: Graphs depict log2 expression counts for selected genes involved in the fatty acid oxidation pathway. (D) Glycolysis Pathway Gene Expression: Highlighting the expression levels of genes involved in glycolysis. (C, D) Analyses based on the GSE94438 dataset from RNA sequencing of whole blood cells from TB patients and healthy controls in a household contact study. Error bars represent Mean ± SD. Statistical significance in (C) and (D) was assessed using an unpaired t-test, with p-values denoted as follows: *p<0.05, **p<0.01, ***p<0.001, ****p<0.0001; ns indicates a non-significant result. Illustrations created with www.BioRender.com. (TIF) [file ppat.1012188.s003.tif]

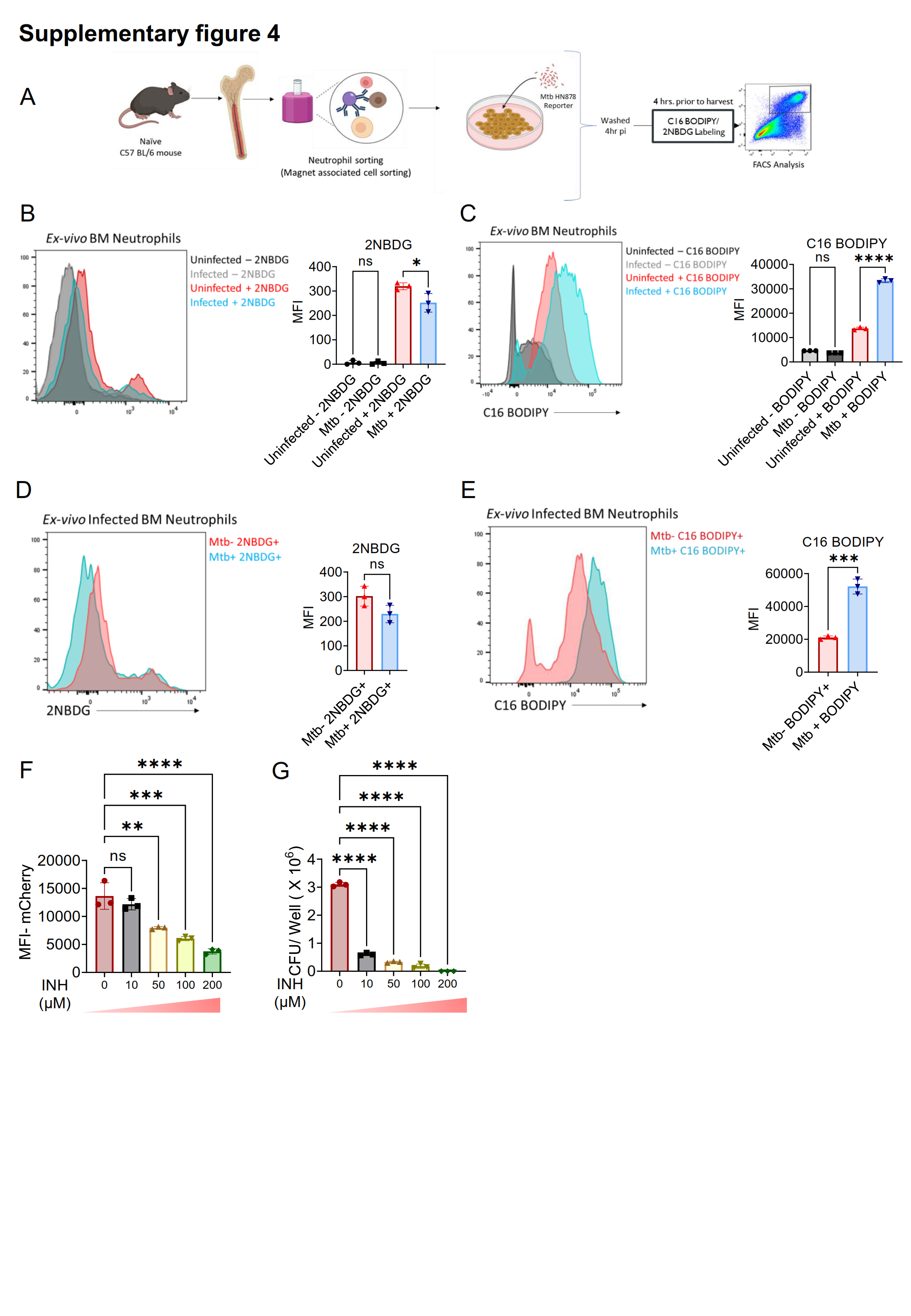

Supplement: S4 Fig — (A) Bone marrow neutrophils from 6–8-week-old BL/6 mice were isolated and infected ex-vivo with Mtb HN878 s-myc’::mCherry at an MOI of 3. After washing to remove extracellular bacteria 4 hours pi, cells were incubated for another 20 hours. The metabolic activity of neutrophils post-infection is assessed using fluorescent glucose and fatty acid analogs. Both infected and uninfected neutrophils were then labeled with C16-BODIPY or 2-NBDG 4 hours before harvesting and analyzed via flow cytometry. (B) 2-NBDG Uptake: Flow cytometry histogram and MFI graph comparing 2-NBDG uptake (15μM) in uninfected versus infected neutrophils, indicating changes in glucose metabolism post-infection. (C) C16-BODIPY Labeling: Histogram and MFI graph showing fatty acid analog C16-BODIPY labeling (25μM) in uninfected versus infected neutrophils, reflecting fatty acid metabolism alterations upon infection. (D) Flow cytometry histogram and MFI graph of 2-NBDG in neutrophils with and without bacteria following ex-vivo challenge with Mtb HN878. (E) Histogram and MFI graph of C16-BODIPY in Mtb+ (infected) and Mtb- (uninfected) neutrophils upon ex-vivo challenge with Mtb HN878. Bone marrow neutrophils from BL/6 mice were infected with Mtb HN878 smyc’::mCherry, SSB-GFP at MOI-3. The cells were washed to remove extracellular bacteria 4 hours pi and were analyzed 24 hours pi. They were treated with increasing concentrations of isoniazid (INH) and MFI of mCherry (F) and neutrophil bacterial burden by CFU was enumerated (G). Sample size n = 3 replicates per group. Error bars denote Mean ± SD. Statistical analyses performed include ordinary one-way ANOVA for (B, C, F, G), with subsequent Tukey’s multiple comparison tests, and unpaired t-tests for (D, E). Significance is denoted as follows: *p<0.05, **p<0.01, ***p<0.001, ****p<0.0001; ns indicates a non-significant difference. Illustrations created with www.BioRender.com. (TIF) [file ppat.1012188.s004.tif]

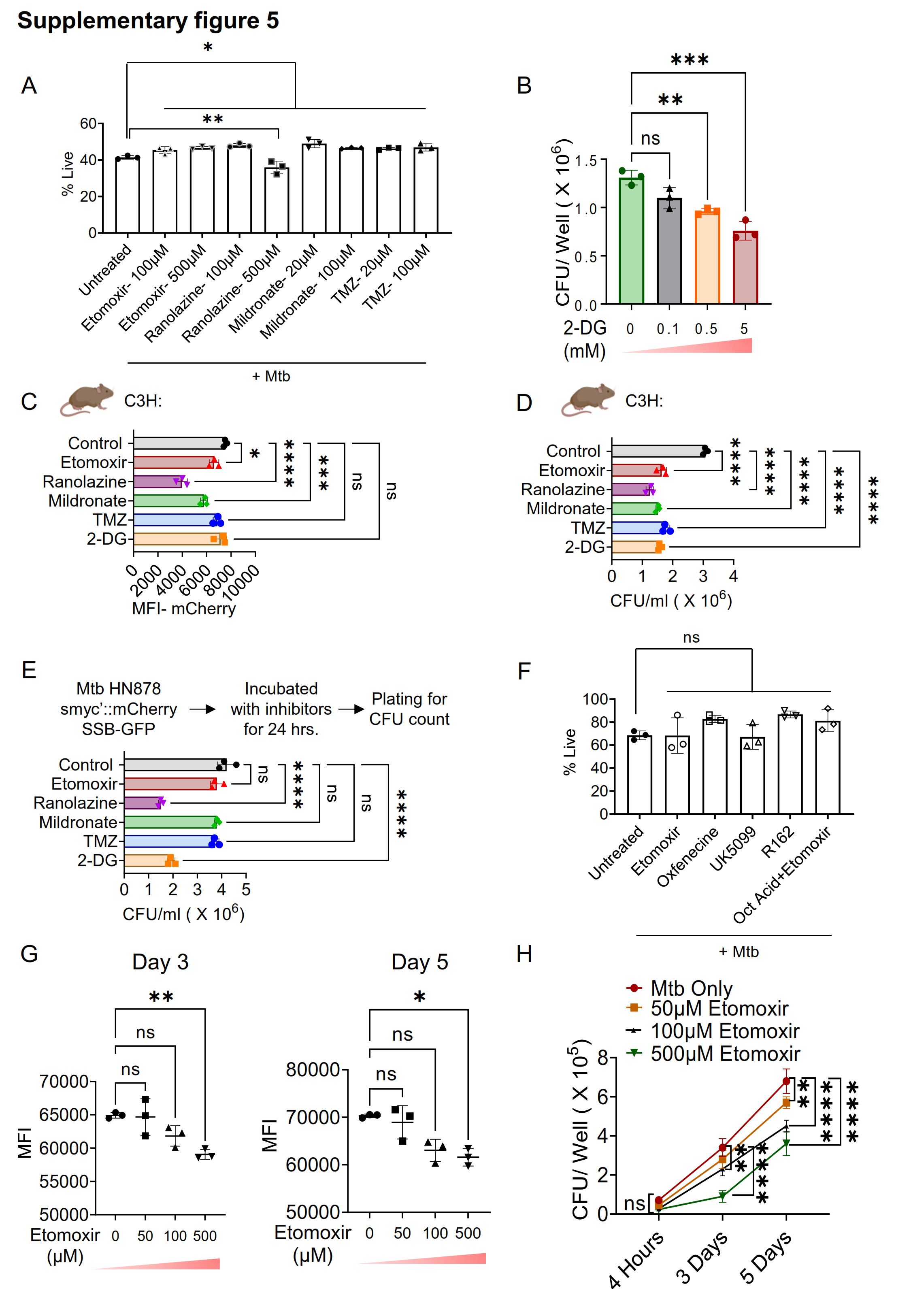

Supplement: S5 Fig — (A) The cytotoxicity of neutrophils treated with fatty acid oxidation inhibitors at indicated concentrations (Etomoxir, Ranolazine, Mildronate, and TMZ) was assessed at 24 hours pi by flow cytometry, reporting the percentage of cells that are fixable viability dye (FVD) negative. (B) Neutrophils were treated with either 0.1mM, 0.5mM, or 5mM 2-Deoxy Glucose (2DG), followed by measurement of bacterial burden by CFU counts at 24 hours pi. (C) Bone marrow neutrophils from C3H mice were infected with Mtb HN878 smyc’::mCherry, SSB-GFP ex-vivo. Cells were washed 4 hours pi to remove extracellular bacteria and were incubated further for 20 hours. Neutrophils were treated with 200μM ETO/ 100μM Ran/ 100μM Mil/ 100μM TMZ/ 0.5mM 2-DG and 24 hours pi, MFI of mCherry was assessed. (D) Bacterial burden in these neutrophils from (C) were enumerated by CFU counts. (E) CFU graph depicting the inhibitory effects of various concentrations of metabolic inhibitors, including 500μM Etomoxir, 100μM Mildronate, 100μM TMZ, 500μM Ranolazine, and 5mM 2-DG on Mtb broth culture. (F) Flow cytometry analysis of neutrophil cell death post treatment with mitochondrial metabolism inhibitors (Etomoxir, Oxfenicine, UK5099, R162, as used in Fig 3F) or with Etomoxir after supplementation with the medium-chain fatty acid, Octanoic acid, at 24 hours pi (% FVD- cells). (G) Bone Marrow Derived Macrophages (BMDM) were harvested from naïve BL/6 mice and infected with an MOI-3 of Mtb HN878 reporter. Graphs displaying the MFI of Mtb HN878 smyc’::mCherry at 3 and 5 dpi, compared to untreated controls. (H) Bacterial load in BMDMs at various time points post infection was determined by CFU counts. The experiments were conducted with n = 3 replicates per group, representative of two experiments. Error bars indicate Mean ± SD. Statistical analyses were performed using one-way ANOVA for (A-G), and two-way ANOVA for (H). Tukey’s multiple comparison tests were used post hoc to determine significance: *p<0.05; **p<0.01; [file ppat.1012188.s005.tif]

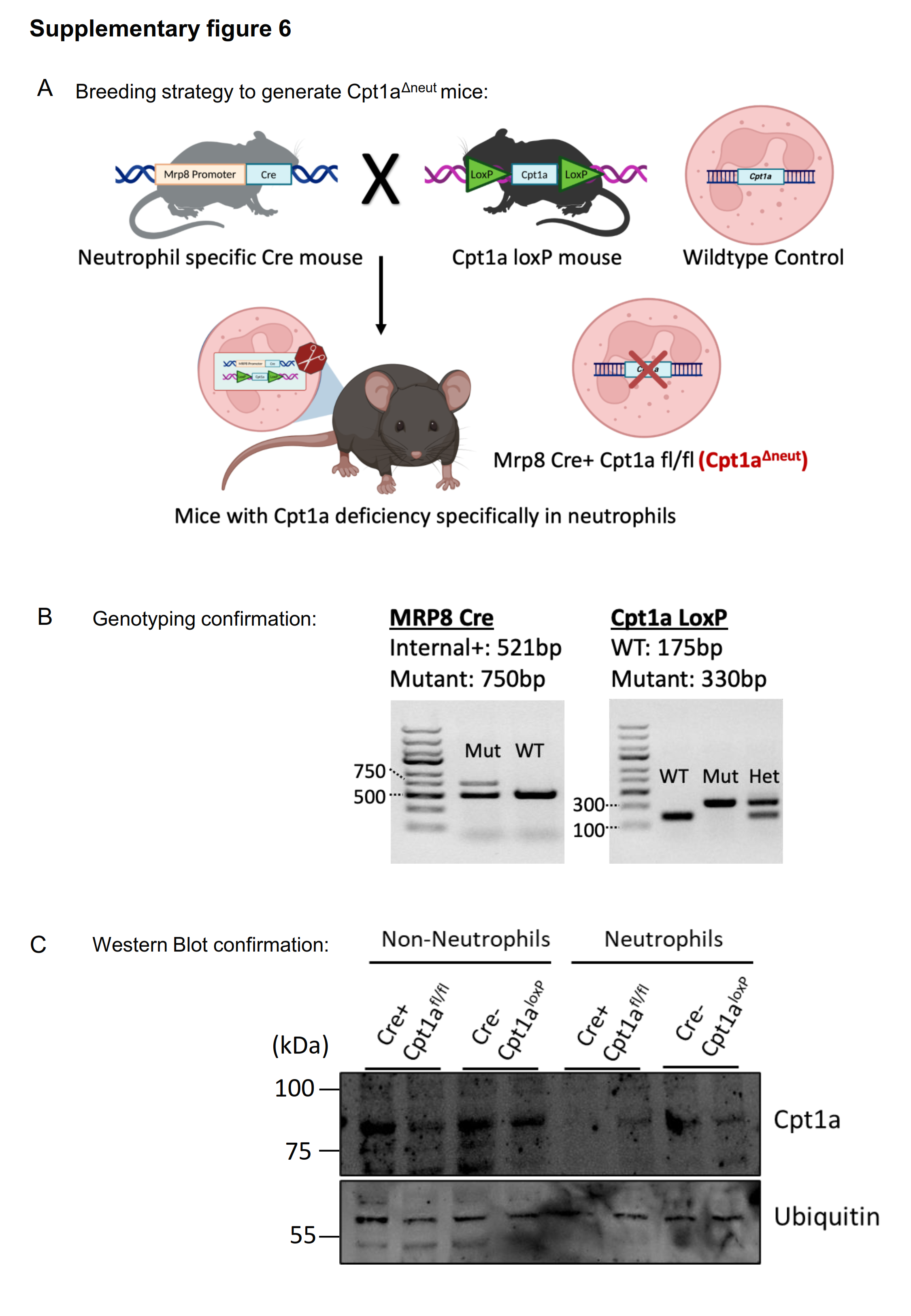

Supplement: S6 Fig — (A) Infographic showing the generation of neutrophil-specific Cpt1a knockout mice (Mrp8-cre Cpt1afl/fl, designated as Cpt1aΔneut). (B) Genotyping to validate the presence of MRP8-Cre (left) and Cpt1a loxP (right) genes in Cpt1aΔneut mice. (C) Western blot analysis to confirm the absence of Cpt1a (Size: ~86 kDa) in Mrp8 Cre+ Cpt1a fl/fl mouse bone marrow neutrophils, isolated by magnetic sorting and presence of Cpt1a in Mrp8 Cre- Cpt1a loxP neutrophils and other bone marrow cells in Cre+ and Cre- mice (top panel) with housekeeping gene Ubiquitin (bottom panel). Illustrations created with www.BioRender.com. (TIF) [file ppat.1012188.s006.tif]

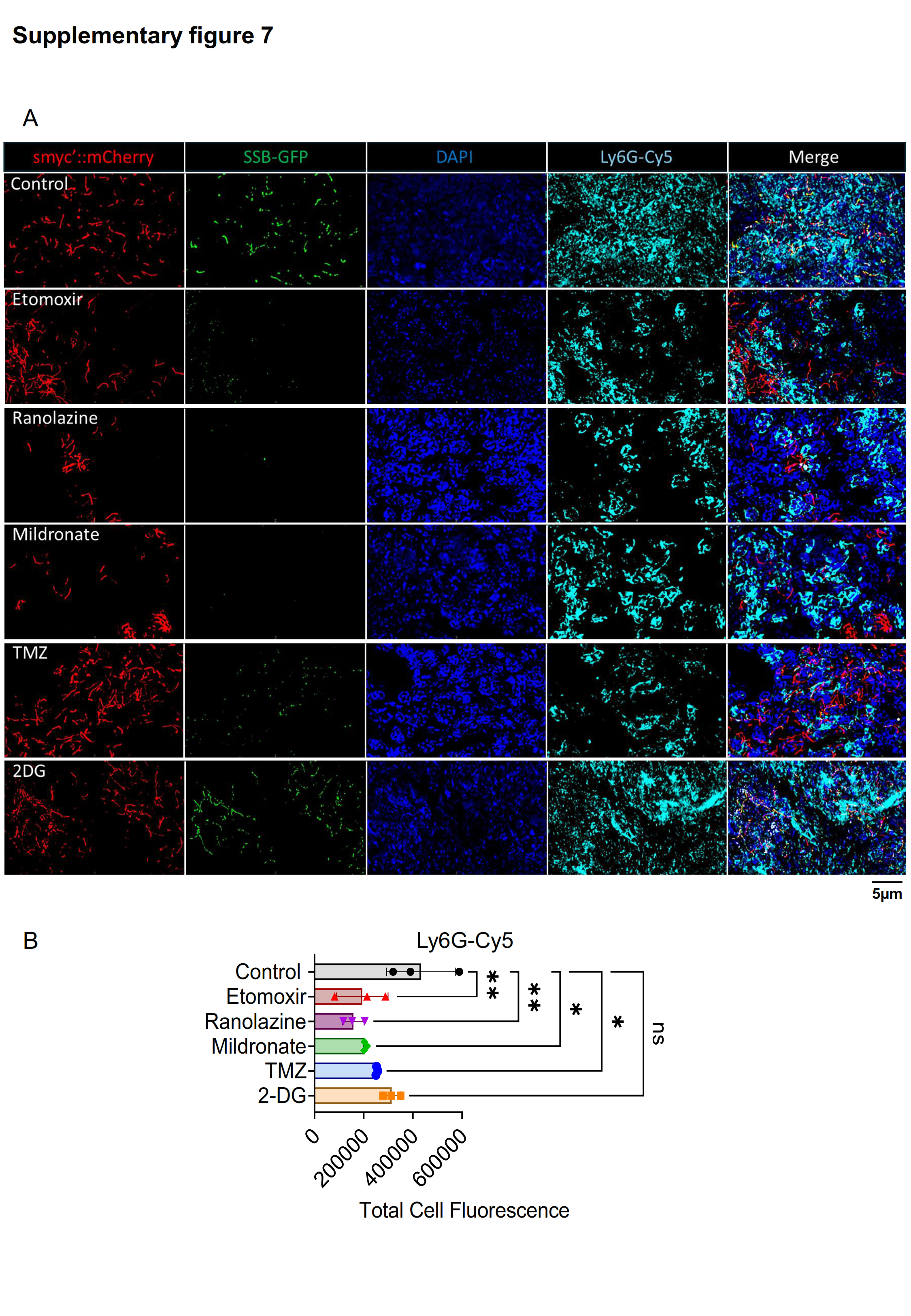

Supplement: S7 Fig — (A) Confocal microscopy images of lung sections (top to bottom) from control, etomoxir, ranolazine, mildronate, TMZ, and 2-DG treated mice (from Fig 4A). Mtb is visualized using smyc’::mCherry, SSB foci are visualized by GFP, neutrophils are stained with Ly6G-Cy5, and nuclei are counterstained with DAPI. (B) Total cell fluorescence of Ly6G staining was quantified by Image J. Representative image of n = 3 mice / group, 3 fields of view per mouse, 200–500 bacteria per image. Error bars indicate Mean ± SD; Scale- 5μm. Statistical analysis was performed using one-way ANOVA (B) with Tukey’s multiple comparison test for significance: p < 0.05, *p < 0.01, **p < 0.001, ***p < 0.0001; ns, not significant. (TIF) [file ppat.1012188.s007.tif]

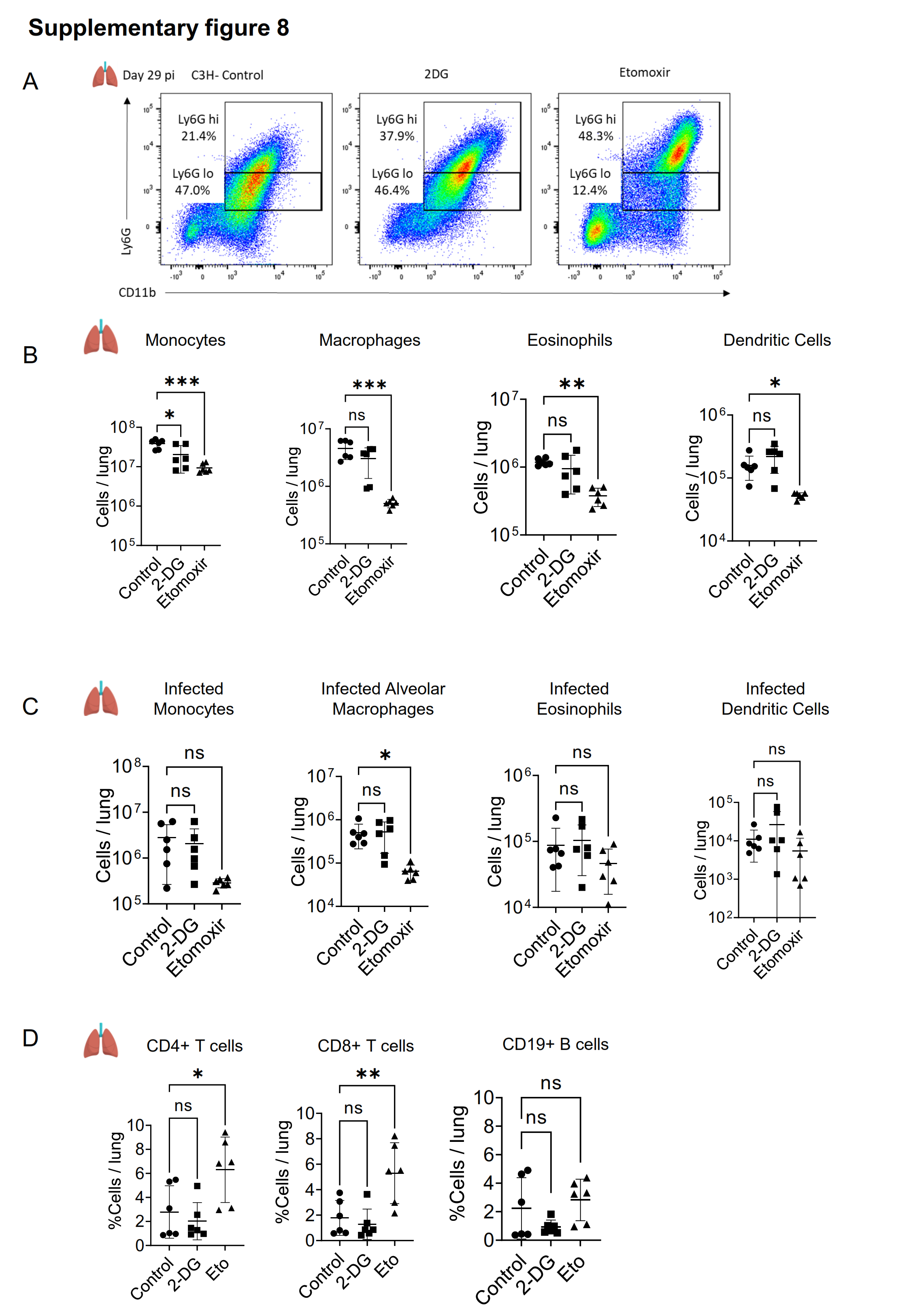

Supplement: S8 Fig — (A) Representative flow cytometric plots display the Ly6Ghi and Ly6Glo/dim neutrophil profiles from BL/6 and C3H untreated control, 2-DG-treated, and Etomoxir-treated mice on 29 dpi with Mtb HN878 smyc’::mCherry, SSB-GFP. (B) Graphs depict the counts of various live cell types, including monocytes (Live CD11b+ Ly6G- Ly6C+), macrophages (Live CD11b+ Ly6G- Ly6C- F4/80+), eosinophils (Live CD11b+ Ly6G- Ly6C- F4/80- SigF+), and dendritic cells (Live CD11b- Ly6G- Ly6C+ SigH+), in the lungs of untreated control, 2-DG-treated, and Etomoxir-treated mice at 29 dpi. (C) Total numbers of infected immune cells (smyc’::mCherry+), including monocytes, macrophages, eosinophils, and dendritic cells, are compared among Control, 2-DG, and Etomoxir-treated mice at 29 dpi. (D) The percentage of live CD4+ T-cells (Live CD11b- CD19- CD3+ CD4+), CD8+ T-cells (Live CD11b- CD19- CD3+ CD8+), and CD19+ B-cells (Live CD11b- CD3- CD19+) within the lungs of untreated controls, 2-DG, and Etomoxir-treated mice are shown at 29 dpi. Sample size n = 6 mice per group, representative data from two experiments. For (H), n = 3 mice/ group, in triplicates. Error bars indicate Mean ± SD. Statistical analysis was performed using one-way ANOVA with Tukey’s multiple comparison tests for significance: *p<0.05, **p<0.01,***p<0.001, ****p<0.0001; ns denotes non-significant differences. Clip art/Images within figure panels were created with www.BioRender.com. (TIF) [file ppat.1012188.s008.tif]

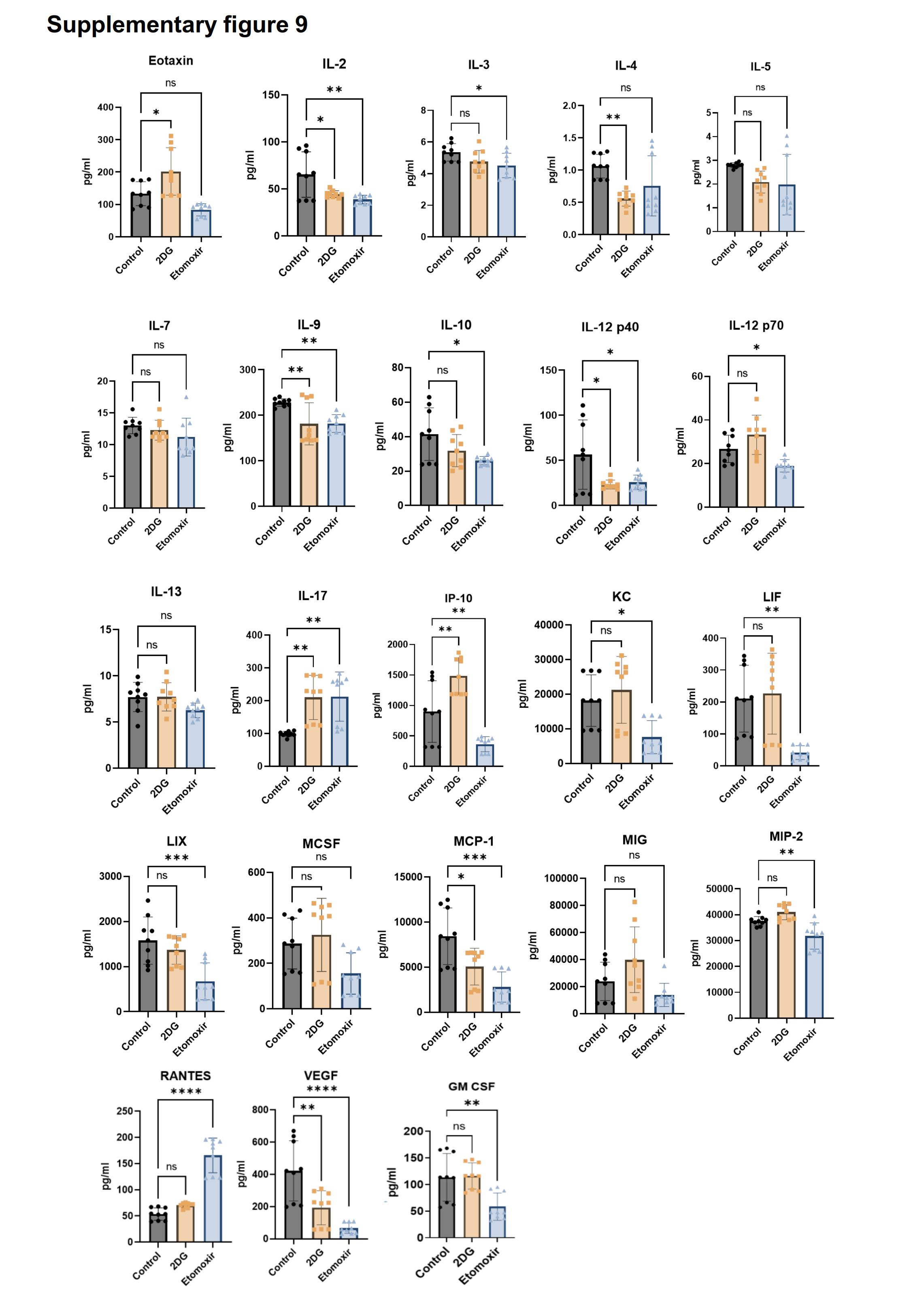

Supplement: S9 Fig — Cytokine levels were quantitatively analyzed in lung homogenates from mice treated with 2-DG or Etomoxir in comparison to untreated controls. The graphs present a comparison of various cytokines, including Eotaxin, IL-2 to IL-17, IP-10, KC, LIF, LIX, MCSF, MCP-1, MIG, MIP-2, RANTES, VEGF, and GM-CSF, measured in picograms per milliliter (pg/ml). Sample size n = 3 mice per group, in triplicates. Error bars represent Mean ± standard deviation (SD). Statistical analyses were conducted using one-way ANOVA with Tukey’s multiple comparison tests to determine significance. The stars indicate levels of statistical significance with *p<0.05, **p<0.01, ***p<0.001, ****p<0.0001; ’ns’ denotes non-significant differences. (TIF) [file ppat.1012188.s009.tif]

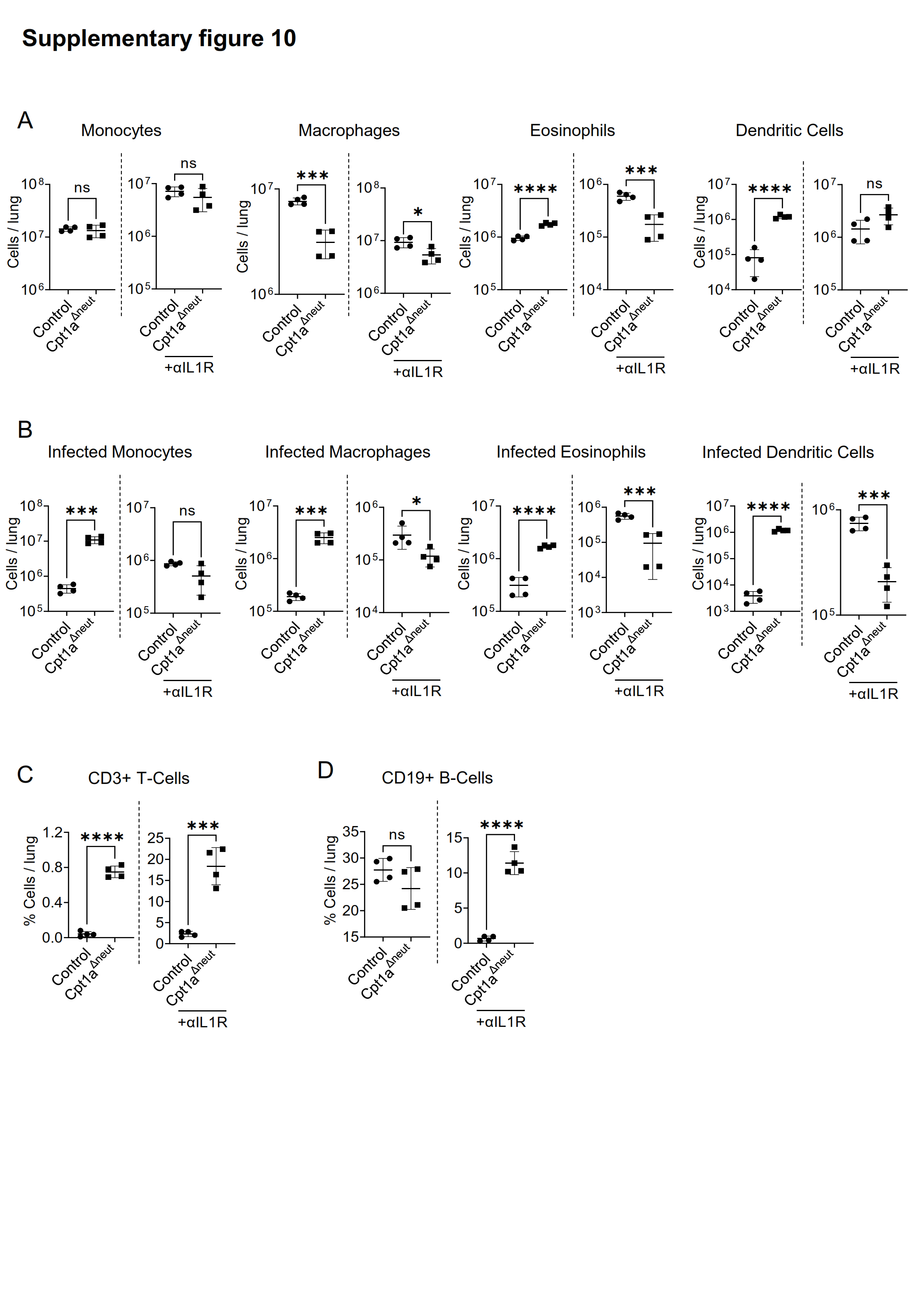

Supplement: S10 Fig — (A) Enumeration of total numbers of live monocytes (Live CD11b+ Ly6G- Ly6C+), macrophages (Live CD11b+ Ly6G- Ly6C- F4/80+), eosinophils (Live CD11b+ Ly6G- Ly6C- F4/80- SigF+), and dendritic cells (Live CD11b- Ly6G- Ly6C+ SigH+), in the lungs of littermate controls and Cpt1aΔneut mice (left) and α-IL1R-treated controls and Cpt1aΔneut mice (right) infected with Mtb HN878 smyc’::mCherry at 29 dpi. (B) Quantification of the total number of infected (smyc’::mCherry+) monocytes, macrophages, eosinophils, and dendritic cells in the lungs of control and Cpt1aΔneut mice (left) and α-IL1R-treated control and Cpt1aΔneut mice (right) at 29 dpi. (C) Proportion of total CD3+ T-cells (Live CD11b- CD19- CD3+) and (D) CD19+ B-cells (Live CD11b- CD3- CD19+) in control and Cpt1aΔneut mice (left) and α-IL1R-treated control and Cpt1aΔneut mice (right) at 29 dpi. Sample size n = 4 mice per group. Error bars indicate Mean ± SD, are from one experiment. Statistical analysis was performed using unpaired t-tests for significance: p < 0.05, *p < 0.01, **p < 0.001, ***p < 0.0001; ns, not significant. (TIF) [file ppat.1012188.s010.tif]
