# Supplementary material for: Fatty acid metabolism in neutrophils promotes lung damage and bacterial replication during tuberculosis
Source: PLoS Pathog. 2024 Oct 4;20(10):e1012188. doi: 10.1371/journal.ppat.1012188 (PMC11482725; doi:10.1371/journal.ppat.1012188)
Supplement: Attachment — Submitted filename: FAO Manuscript_Point by point response 8.20.24.pdf [file ppat.1012188.s013.pdf]

ALBANY MEDICAL COLLEGE

ALBANY MED Health System

Aug 18, 2024

To,

Helena Boshoff, Ph.D.
The Editor,
PLOS Pathogens

Subject: "Fatty acid metabolism in neutrophils promotes lung damage and bacterial replication during tuberculosis." (PPATHOGENS-D-24-00766).

Dear Dr. Boshoff,

We are writing in response to the peer review for our manuscript, "ID: PPATHOGENS-D-24-00766," titled "Fatty acid metabolism in neutrophils promotes lung damage and bacterial replication during tuberculosis," submitted to PLOS Pathogens. We sincerely thank you and the reviewers for the time and effort spent reviewing our manuscript and giving us the opportunity to revise it.

We have conducted the additional experiments suggested by the reviewers to further strengthen the results presented. The revised manuscript now includes data from in vivo infection of the conditional knockout mice we generated, as well as experiments inhibiting fatty acid metabolism in another susceptible mouse model. We have also expanded the discussion to contextualize our findings with previous publications and recent literature in the field. Additionally, we have clarified the methodology and experimental setups to improve the interpretation of our results. Each of the reviewers' concerns has been addressed in detail in our point-by-point response, which is included with this letter. All changes made to the main text of the manuscript are highlighted in blue.

We believe our manuscript now meets the esteemed standards of PLOS Pathogens for publication. We sincerely appreciate your consideration of our manuscript.

We look forward to hearing from you.

Best regards,

Bibhuti B Mishra, Ph.D.
(mishrab@amc.edu)

Point by point response

Reviewer #1: -

Comment: How were the SSB-GFP foci quantified for the graphs shown in Fig. 1C and Fig. 4E? In particular, is one data point from one mouse? It is unclear from the figure legend or the methods how this quantification was conducted. Particularly given the range in the percentage of Mtb that exhibit SSB-GFP foci that can be observed across images, robust quantification requires enumeration of a percentage of SSB-GFP foci from observations across multiple images for one mouse, with one mouse thus providing one data point. Can the authors please clarify.

Response: We appreciate the reviewer's suggestion to clarify the figure legends. In the quantification graphs for **Fig. 1C and Fig. 4E**, each data point represents the mean percentage of SSB-GFP foci, which was determined by averaging the percentage of SSB-GFP foci-containing bacteria from a single field of view. For each mouse, three individual fields of view were used to quantify this percentage. To generate the mean, a total of three mouse lungs (n=3) were imaged to calculate the frequency of replicating bacteria. Each data point hence represents one mouse. We have now clarified this information in both the figure legends and the methods section (Methods: Lines 783- 785; **Fig. 1E**, Lines 1020-1022; **Fig. 4E**- Lines 1111-1112).

Comment: Related to the above, it is overall unclear how the determination regarding Mtb replication state was made for the various host cell types (lines 145-156). Were these conclusions made from microscopy quantification (and if so, how exactly was this done; see questions above), or from flow cytometry (Fig. S2A)?

Response: We apologize for not clearly stating this in our manuscript. The determination of the replication state of Mtb in various host cells was conducted through microscopy analysis of cytospins prepared from lung cells (see **Fig. 1F, Supplementary Fig. 2A-C**). Different cell types were sorted from Mtb-infected mouse lungs based on their CD11b, CD64, Ly6G, and Siglec F staining. Cytospins were then prepared from these sorted cell fractions, and the presence of bacteria, including replicating bacteria (identified by SSB-foci), was assessed in the microscopy images as described above. Cytospins were prepared from the lungs of three mice, and the frequency of replicating bacteria was calculated by imaging three different fields of view per mouse. We have now clarified this information in both the figure legends and the methods section (Methods: Lines- 770-775, 787-795).

Comment: The flow cytometry plot in Fig. S2A does not robustly show separation of SSB-GFP positive versus negative Mtb. And indeed, the one bacterium shown as an example for each class in the cytospin in Fig. 1F raise concerns regarding the ability of flow cytometry to properly distinguish SSB-GFP positive versus negative Mtb – the one Mtb bacillus shown as "mCherry+ GFP lo" actually possesses an SSB-GFP focus and should be considered as being in a replicating state. SSB-GFP signal is present to some degree in all bacteria regardless of DNA replication status, with the localization of signal that differs when DNA replication is active, as the authors note.

Response: We acknowledge the confusion caused by our sorting images in establishing flow cytometry to accurately differentiate between SSB-GFP+ and SSB-GFP- bacteria. However, we want to emphasize that the primary focus of this paper is not on developing a methodology for studying replicating and non-replicating bacteria via flow cytometry. To avoid further confusion and to align with established literature, we have now exclusively used microscopy-based analysis to assess the bacterial replication state. These data were now shown in **Fig. 1C, Fig. 4E**.

To demonstrate the presence of replicating bacteria within neutrophils, we sorted infected neutrophils based on their smyc'::mCherry positivity and identified bacteria containing SSB-GFP foci within Ly6G+ cells through microscopy (**Fig. 1F**). For our *ex-vivo* experiments (**Fig. 3**), we used CFU to measure bacterial replication, while for our *in-vivo* experiments (**Fig. 4**), we used both CFU and microscopy, as recommended by the reviewers, to quantify bacterial replication.

Comment: A control flow cytometry sorting using Mtb in known states of replication versus not (i.e. from in vitro Mtb experiments in defined conditions), with appropriate microscopy comparison, is needed to establish flow cytometry as a means for successful separation of SSB-GFP positive versus negative Mtb. In the absence of robust establishment that flow cytometry is able to properly distinguish SSB-GFP-positive from SSB-GFP-negative Mtb, analysis should be conducted via microscopy only, with appropriate numbers of images/animals, as noted above.

Response: We appreciate the reviewers' suggestion. As mentioned earlier, we have now concentrated our analysis of bacterial replication solely on a microscopy-based approach. We do not aim to establish flow cytometry as a method for distinguishing between SSB-GFP-positive and SSB-GFP-negative Mtb, as this would detract from the primary goal of our study: demonstrating that neutrophils serve as a permissive niche for Mtb in susceptible mice and they rely on fatty acid metabolism to support intracellular bacterial growth.

Comment: The exacerbation of Mtb infection with 2-DG treatment, versus the improved control of Mtb infection by FAO inhibition, has previously been shown by various groups. In this context, the effect of these treatments on other key immune cell types for Mtb (e.g. alveolar versus interstitial macrophages) have also been described. It is important that the authors acknowledge prior literature on this and incorporate that information into their conclusions and discussion.

Response: We apologize for not adequately discussing previous studies. We have now discussed this study by Pacl et al, Nature Communications 2023, PMID: 37673914 and elaborated on this section in the revised manuscript in lines- 569-574.

Comment: Indeed, as the authors note, "ETO administration reduced the infiltration of monocytes, macrophages, eosinophils, and plasmacytoid dendritic cells (Fig. S6B-now S8B), with a significant decrease in the number of infected alveolar and interstitial macrophages (Fig. S6C-now S8C)". How do the authors differentiate the relative contributions of the alterations to the different host immune cell types to the in vivo outcome with Mtb infection observed?

Response: We appreciate the reviewers' emphasis on this important point. First, we would like to highlight that in our infection model, we observed a significant increase in neutrophil infiltration and a higher proportion of infections within neutrophils compared to other immune cells (**Fig. 1D-G, Supplementary Fig. 1D, E**). This finding underscores the critical importance of focusing on neutrophil infiltration and replication of bacteria within these cells. Regarding the effect of ETO administration, our results showed a general decline in various myeloid cells, including both infected neutrophils and macrophages. While we acknowledge the possibility of an off-target effect of ETO on immune cell recruitment during Mtb infection, we would like to note that in our Cpt1a$^{\Delta neut}$ mice, we observed a reduction in the number of infected neutrophils, accompanied by an increase in the number of infected monocytes and macrophages (**Fig. 6F, Supplementary Fig. 10B**). These observations suggest that inhibiting neutrophil recruitment, whether through ETO administration or the genetic deletion of Cpt1a in neutrophils, could potentially result in improved outcomes, as indicated by the altered cellular distribution of Mtb and its association with TB susceptibility.

Comment: Fig. 5A, lines 383-384: The authors state that there is a "dose-dependent inhibition of neutrophil migration by ETO", but the data shown do not in fact demonstrate any dose-dependence. This should be clarified, and the conclusions adjusted accordingly.

Response: In response to the reviewer's suggestions, we repeated the transwell experiment using bone marrow-derived macrophages from BL/6 mice and have replaced the previous data with the updated results in **Fig. 6G**. Interestingly, in response to BL/6 macrophages, we observed a dose-dependent neutrophil migration induced by Etomoxir.

Comment: The materials and methods and the figure legends would benefit from added details, as it is currently difficult in several instances for a reader to figure out what is being shown/how an assay/quantification was performed. To list a few examples:

Response: We appreciate the reviewer's feedback regarding the need for additional details in the materials and methods section as well as the figure legends. In response, we have provided further information where necessary to clarify the procedures and data presentation. We hope these revisions will enhance the reader's understanding of the manuscript.

Comment: It is not clear how quantification in Fig. 1G was performed. How are differences in total number of cells in an image accounted for? What does one data point in the graph represent? One mouse? For all graphs, it should be made clear what data is being presented.

Response: To clarify, for our neutrophil staining in **Fig. 1G**, the total cell fluorescence of Cy5 staining was quantified by calculating the fluorescence intensity from 25 different regions of interest within each field of view, and these values were averaged to obtain a single value per field of view. Each data point in the graph represents one mouse. We have now included this clarification in the figure legends and methods section. For the analysis, 3 images were taken from each mouse lung, with a total of 3 different mice (n=3-4, as mentioned in the figure legends) analyzed (Lines 775-785). To further aid understanding, we have provided the quantification method along with raw values from one randomly selected image for both BL/6 and C3H mice as a part of the provided supplementary information (**Table 1**).

Comment: In Fig. 2G, the figure currently only says "WB". Unclear why the sentence after states sample size for RNA sequencing?

Response: We acknowledge that this might be confusing for the reader. We have now added more space and shifted the sample size and other information to the next line in each figure legend to improve clarity in the manuscript. (Lines 1058-1059).

Comment: In Fig. S2D, information regarding how many animals were in each infection set/time point needs to be added.

Response: We have now highlighted this in our figure legend. Line 1428: and n=4 rabbits per group for **Supplementary Fig. 2D**.

Comment: In Fig. 4D, how was "% necrotic area" determined? How many animals were in each treatment set and how many images/animal were quantified?

Response: The images shown are full organ scans of H&E-stained lungs (Scale- 1mm: **Fig. 4D, 6B**). Lesion areas were defined as regions of the lung occupied by inflammatory infiltration, while areas with minimal or no inflammation and open airways were categorized as uninvolved region. The total area of inflammation was then expressed as a percentage of the whole lung area. We used ImageJ software to select lesions, with the "Analyze" plug-in to quantify the area of each lesion, which was then summed to calculate the total area occupied by inflammatory lesions. Three mouse lungs were quantified in each group to assess statistical significance. The quantification methods have now been included in the methods section (Lines: 803-810)

Comment: In Fig. 4E, no key is provided for what the different colors in the images represent.

Response: We thank the reviewer for pointing this out. We have now added an infographic to the main figure to better clarify the microscopy images. Additionally, we have referred to this in our figure legends (Lines 1111-1112): Visualization and quantification of replicating bacteria (GFP+ on mCherry+ bacilli) in lung sections from infected untreated and inhibitor-treated C3H mice at 29 dpi). Blue: DAPI, Mtb smyc'::mCherry (red), SSB-GFP (green).

Comment: In Fig. 5A, the main text and figure legend state incubation was for 20 hours, while the figure itself states 12 hours.

Response: We apologize for our oversight in the infographic for **Fig. 5A**. We have corrected this discrepancy and added new data for bone marrow-derived macrophages from naïve BL/6 mice.

Reviewer #2:

Comment: The entire manuscript is largely relied on various inhibitors and in vitro studies. In the revision, authors have generated a mouse model that specifically lack Cpt1a (a key enzyme regulating fatty acid oxidation) in neutrophils. This is a great genetic approach to test their hypothesis. However, authors only utilized bone marrow neutrophils isolated from these mice for in vitro studies. Although the data are in line with other experiments utilizing inhibitors, it would be essential and critical for them to test whether fatty acid metabolism in neutrophils promotes Mtb infection in vivo using this mouse model. This experiment will be highly valuable. The authors have the resource to perform this experiment.

Response: We thank the reviewers for acknowledging our genetic approach in testing our hypothesis. While we initially faced challenges in breeding and obtaining enough knockouts for inclusion in the previous version of the manuscript, we have now included the available data in **Fig. 6A-D**. We are pleased to report that the data from our $Cpt1a^{\Delta neut}$ mice are consistent with our earlier observations using bone marrow-derived neutrophils from these mice, where we observed a marginal yet significant reduction in bacterial growth within these cells. In the new *in-vivo* experiments, we also observed a reduced bacterial burden and diminished neutrophil infiltration in the lungs of these mice compared to littermate controls.

Comment: Regarding the mouse model that I mentioned above, in Figure 3G, they only showed the genotyping results. qPCR or western blot data are lacking to confirm Cpt1a is not expressed in neutrophils in those mice.

Response: We thank the reviewer for bringing this to our attention. We have now included these data in **Supplementary Fig. 6C**.

Comment: In Figure 2G, the western blot showing here is concerning, given the level of b-actin in Mtb-infected neutrophils at 24h is significantly less compared to other samples. This implicates that the amounts of protein loaded to the gel are very different. It is also possible that the expression of b-actin in neutrophils was altered after Mtb infection at 24hours. If this is the case, other housekeeping genes will be needed to use as the loading control. The figure legend of this panel is over-simplified without any detailed information.

Response: We thank the reviewer for this observation. Although we ensured that equal amounts of total protein were loaded during the Western blot analysis, we now recognize that β-actin levels may be affected by infection. Therefore, we repeated the experiment using ubiquitin as our loading control. Unlike β-actin, ubiquitin levels appeared unaffected by infection, making it a more reliable control. While the overall conclusions from the Western blot analysis remain unchanged, we have updated the figure legend with this pertinent information (Lines- 1058-1059).

Reviewer #3:

Comment: There is a disconnect in some of the logic through the paper that should be addressed to better support the authors conclusions. First, the RNAseq analysis from neutrophils in B6 v C3H found that FAO was increased in C3H but not B6. No real metabolic analysis was done on these cells and RNA expression is not always indicative of actual metabolic output.
Furthermore, it seems all neutrophils were analyzed so some discussion on how these transcriptional/metabolic changes differ between infected and uninfected neutrophils should be at the very least addressed as a caveat (ie do these neutrophils NEED to be infected to drive this metabolic shift?).

Response: We agree with the reviewer that changes in gene expression may not necessarily reflect the metabolic alterations occurring within cells during infection. We acknowledge that the absence of metabolic analyses is a limitation of our study. However, using fluorescent glucose and fatty acid analogs, we demonstrated that purified neutrophils show a higher propensity for fatty acid utilization, with Mtb-infected cells notably taking up more fatty acids compared to uninfected cells (**Supplementary Fig. 4A-E**).

Traditionally, chemical inhibitors of rate-limiting enzymes have been used to determine whether cells rely on fatty acid oxidation or glucose metabolism. Not only have we demonstrated this using multiple chemical inhibitors, but we have also generated a conditional knockout mouse strain specifically lacking carnitine palmitoyl transferase (Cpt1a), the rate-limiting enzyme of mitochondrial fatty acid oxidation. This allowed us to demonstrate that neutrophils utilize fatty acid metabolism in response to Mtb infection, which plays a key role in TB pathogenesis.

Furthermore, using cell lysates from Mtb-infected neutrophils, we observed increased Cpt1a expression in infected neutrophils compared to uninfected cells ex vivo. While we acknowledge that this method does not differentiate between infected and uninfected cells within the same sample, it does show that Mtb challenge induces Cpt1a expression in neutrophils (Fig.2G).

As suggested, we have now addressed these limitations in the discussion section of the revised manuscript. We recognize that further studies and metabolic analyses are crucial to fully understand the role of fatty acid oxidation during Mtb infections, and we aim to explore these questions in our future research (Lines- 608-620).

Comment: Along these lines it is a little surprising that that all ex vivo work is done in B6 neutrophils that did not show increased FAO and not C3H neutrophils. This seems like a missed opportunity to directly validate their in vivo studies ex vivo in a comparative way that would more fully support the conclusions.

Response: We thank the reviewer for highlighting this important concern. In our early ex vivo studies, we observed that bone marrow neutrophils from BL/6 and C3H mice behave very similarly when subjected to *ex-vivo* infection. Our previous research indicated that the difference in susceptibility between BL/6 and C3H mice is attributed to increased neutrophil influx into the lungs and higher bacterial burden, rather than differences in the intrinsic properties of their neutrophils in terms of bacterial uptake or support for bacterial replication (Mishra et al, Nature Microbiology, 2017 PMID:28504669). Therefore, we conducted all our *ex-vivo* studies using BL/6 mouse neutrophils. We acknowledge that we should have addressed this in the manuscript. In response to the reviewer's suggestion, we inhibited fatty acid oxidation in C3H mouse neutrophils using a range of chemical inhibitors and observed results like those obtained with BL/6 mouse neutrophils (**Supplementary Fig 5C, D**).

Comment: There are key concerns about the in vivo inhibitor experiments that globally block FAO and glycolysis in vivo. Given the importance of these metabolic pathways in a range of cell types Mtb interacts with, some shown here by the authors and previously published by others, it is hard to conclude from this experimental setup that blocking these metabolic pathways is specifically due to changes in neutrophils. It could be these inhibitors are altering Type I IFN and other cytokines in the macrophages that then alter neutrophils recruitment and function during infection. Given the authors made a neutrophil specific CPT1 mouse it seems leveraging this in one of the other susceptible mouse models (i.e. Nos2 or IL1R) or leveraging recently published SP140 mice that phenocopy many aspects of C3H mice on the B6 background are really needed to support the authors conclusions. I understand that this is a large undertaking, but if the authors really want to make the conclusions that neutrophil specific FAO is critical in vivo as written it seems to be very important to not globally block these metabolic pathways which may have many pleotropic effects on infection and disease progression.

Response: We thank the reviewer for the valuable suggestion. We recognize the insights that could be gained from generating neutrophil-specific Cpt1a knockouts in susceptible mice. However, due to time constraints and the challenges we have encountered in breeding and generating our Cpt1a$^{\Delta neut}$ mice, we were unable to produce these mice in various susceptible backgrounds.

Given the essential role of IL-1R signaling in both C3H and Sp140 KO mice (PMID:34151776), we hypothesized that selective blockade of IL-1R in our conditional Cpt1a knockout mice could mimic many aspects of the pathogenesis mechanisms observed in susceptible mice. To test this, we employed two different approaches.

First, we treated Cpt1a$^{\Delta neut}$ mice with an anti-IL-1R monoclonal antibody and compared the bacterial burden, neutrophil influx, and lung pathology to that of littermate controls treated similarly. The Cpt1a$^{\Delta neut}$ mice showed improved lung pathology, reduced bacterial burden, and decreased neutrophil infiltration in the lungs compared to the littermate controls treated with IL-1R. This further underscores the role of neutrophil fatty acid oxidation in the pathogenesis of TB (**Fig. 6A-F**).

Additionally, we treated susceptible IL1R-/- mice with Mildronate, a global fatty acid oxidation inhibitor, and evaluated various disease parameters. Compared to vehicle-treated mice, those treated with Mildronate exhibited reduced lung neutrophil infiltration, lower bacterial burden, and less weight loss, similar to the results seen in the susceptible C3H mouse model. This indicates a common pathogenic mechanism associated with TB susceptibility (**Fig. 5**).

Comment: The transition of the final figure of the manuscript to focus on NETs feels disjointed and a little underdeveloped. Cybb is known to be a key player in driving NET formation (one example: PMID: 17210947). How do the authors explain that their phenotype is not observed in Cybb neutrophils then? This suggests there may be something else distinct between the Pad4 and Cybb neutrophils that is not dependent on NETS. However, without directly quantifying whether NETs are produced, instead of relying on just microbial readouts, it is difficult to interpret and make strong conclusions from these data. I am not convinced these findings are needed given their preliminary state and I may cautiously suggest removing them to keep the focus of the manuscript clearer and more supported by the data. Alternatively, more experiments to support these conclusions and clarify the results are needed.

Response: We thank the reviewer for these insights. We apologize for not clearly discussing the results. The primary aim of the experiment was to determine whether two key neutrophil effector mechanisms—NETs formation and ROS generation—are involved in the improved bacterial control by neutrophils when the fatty acid oxidation pathway is inhibited. While we agree that ROS formation is a crucial step in NETs formation, and Cybb-/- neutrophils are therefore expected not to produce NETs, we cannot entirely rule out the possibility of ROS-independent but Pad4-dependent NETs formation as a potential antimicrobial mechanism that will be followed up in details in future studies. However, in response to the reviewer's suggestion and to maintain the manuscript's focus on neutrophil metabolism, we have now removed the data related to Pad4 and Cybb knockout neutrophils.

Part III – Minor Issues: Editorial and Data Presentation Modifications

Reviewer #1:

Comment:2-NBDG and BODIPY have overlapping spectra with GFP. Since the bacteria used in the experiments shown in Fig. S4 carry the SSB-GFP reporter, is it the case that the 2-NBDG and BODIPY signal were much stronger and essentially the settings were such that SSB-GFP signal was not detected?

Response: We thank the reviewer for bringing this to our attention and apologize for the error in our reporting. In our experiments involving 2-NBDG and C16-BODIPY, we used Mtb HN878 smyc'::mCherry, not the replication reporter as previously stated. We have now corrected this throughout the entire manuscript, including the text and figure legends (Line 242; Lines1455).

Comment: Line 261: It would not appear that there is any Mtb growth observed in the 24 hour infection of neutrophils ex vivo, given the starting inoculum of 6 x 10^6 Mtb/well stated in the methods versus the ~1.25-2.25 x 10^6 Mtb/well obtained from mock-treated cells in Fig. 3B. This sentence should be rephrased.

Response: We understand how the original phrasing could be misleading. We have now removed the phrase "intracellular bacterial growth".

Comment: Is there a reason why Fig. 3B data is shown in a different graph format than Figs. 3C-3E?

Response: In line with the reviewer's suggestion, we have now converted **Fig. 3B** into a bar graph to maintain consistency with the other data in that figure.

Comment: Fig. 3F: Octanoic acid alone improves Mtb growth, which makes it somewhat difficult to draw conclusions regarding the phenotype observed with ETO + octanoic acid. At minimum the authors' conclusion regarding the use of octanoic acid to mitigate the effects of ETO should be tempered.

Response: We concur with the reviewer's observation that octanoic acid alone increased Mtb growth, and when combined with Etomoxir, it resulted in a similar increase in bacterial growth. We interpret these observations as follows: When fatty acids are available as a carbon source, neutrophils may utilize this medium-chain fatty acid for energy in response to Mtb infection. Although Etomoxir blocks the import of acetyl carnitine into the mitochondrial lumen for fatty acid oxidation (FAO), the addition of excess fatty acid can bypass the Etomoxir-mediated suppression of FAO. Furthermore, we cannot exclude the possibility that octanoic acid may be utilized by Mtb for its survival within neutrophils.

To avoid confusion, we have removed the data point for octanoic acid alone and included only the data for the Etomoxir + octanoic acid combination. In our opinion, this adjustment does not alter the overall conclusions drawn from the experimental results in **Fig. 3F**. Additionally, we have rephrased the relevant statement to: "The effect of Etomoxir was reversed by co-treatment with octanoic acid, as shown by an increase in mCherry MFI (Lines 323-324)."

Comment: Are there other effects of Pad4 deletion beyond NET formation?

Response: The revised version of the manuscript does not include any data from Pad4 KO mice. This decision was made in response to Reviewer #3's recommendation to maintain the manuscript's focus on fatty acid metabolism in neutrophils and its role in bacterial control. Therefore, this data is no longer relevant to the current scope of the study.

Comment: "smyc'" should be italicized.

Response: This has now been corrected throughout the manuscript.

Reviewer #2:
Comment: The statistical information is lacking in Figure 4D. It looks like only one sample from each group was used to calculate % necrotic lesion area.

Response: We apologize for not including this information in our earlier submission. We have now added both the statistical data and the method used for calculating the percentage of lesion area (**Fig. 4D,** Lines- 803-810).

Reviewer #3:
Comment: It was not described why for the transwell experiment human macrophages were used as a source of infected cells while mouse neutrophils were then used. It seems some differences between mouse and human could confound these results and it is straightforward to use murine BMDMs or other macrophage lines to directly answer this.

Response: We understand that murine macrophages are more relevant to this study. Following the reviewer's suggestions, we have repeated the experiment, and the new data is now included as part of **Fig. 6G-I**.

Comment: While I understand that ex vivo neutrophils are challenging given how rapidly the die, I do have some concerns around the robustness of the mCherry/CFU experiments in neutrophils given the very short timescale. First, mCherry has a very stable half-life thus seeing a reliable decrease in MFI (even if modest) may be difficult to make reliable conclusions from. Including an antibiotic treatment control in these experiments where you know Mtb is being killed would help alleviate some of these concerns given the short time scale. Alternatively using imaging approaches with the SSB reporter to more quantitatively assess bacterial burden in these cells may be another approach to better support these conclusions.

Response: We thank the reviewer for this insightful comment. We understand the importance of establishing MFI of mCherry as a reliable readout for our *ex-vivo* experiments. To address this concern, we treated our ex vivo sorted neutrophils with increasing concentrations of the antibiotic Isoniazid. This resulted in a steady, dose-dependent reduction in MFI of mCherry, which was corroborated by a reduced bacterial burden as assessed by CFU analysis. These data are now included in the **Supplementary Fig. 4F, G.**
