# Supplementary material for: Fatty acid metabolism in neutrophils promotes lung damage and bacterial replication during tuberculosis
Source: PLoS Pathog. 2024 Oct 4;20(10):e1012188. doi: 10.1371/journal.ppat.1012188 (PMC11482725; doi:10.1371/journal.ppat.1012188)
Supplement: Attachment — Submitted filename: FAO Manuscript_Point by point response 9.16.24.pdf [file ppat.1012188.s014.pdf]

ALBANY MEDICAL COLLEGE
ALBANY MED Health System

Sept 16, 2024

To,

Helena Boshoff, Ph.D.
The Editor,
PLOS Pathogens

Subject: "Fatty acid metabolism in neutrophils promotes lung damage and bacterial replication during tuberculosis." (PPATHOGENS-D-24-00766R1- [EMID:46bdef10faf7384b]).

Dear Dr. Boshoff,

We are writing in response to the peer review for our manuscript, "PPATHOGENS-D-24-00766R1- [EMID:46bdef10faf7384b]", titled "Fatty acid metabolism in neutrophils promotes lung damage and bacterial replication during tuberculosis," submitted to PLOS Pathogens. We sincerely thank you and the reviewers for taking the time to review our manuscript and provide valuable feedback.

In response to the recommendations, we have revised the manuscript by including additional experimental details and specifying sample numbers to enhance the clarity of the study results. Each of the reviewers' concerns has been addressed comprehensively in the point-by-point response accompanying this letter. All changes made to the main text are noted with their corresponding line numbers.

We believe our manuscript now meets the esteemed standards of PLOS Pathogens for publication. We sincerely appreciate your consideration of our manuscript.

We look forward to hearing from you.

Best regards,

Bibhuti B Mishra, Ph.D.
(mishrab@amc.edu)
Laboratory of Tuberculosis Pathogenesis
Albany Medical College, Albany, New York

Point by point response

Reviewer #3:

Reviewer's Comments: This revised manuscript examines how Fatty Acid metabolism in neutrophils contributes to disease severity during Mycobacterium tuberculosis. The authors have added significantly more data with their Cpt KO mouse, as well as addressed lipid metabolism in a susceptible mouse strain. The added data help to support the conclusions more directly and these data will be of great interest to immunologists and mycobacterial researchers. two small changes may further help set the context and support the conclusions:

1. It is unclear from the figure legends how many times each experiment was completed - especially with the new in vivo experiments. This should be made more explicit for readers to assess the reproducibility of the results.

Response to reviewer: We thank the reviewer for highlighting this point. We have now addressed it by including the relevant information in the figure legends:
Line 1161 (Fig. 5), 1181-1182 (Fig. 6), and 1588 (Supplementary Fig. 10).
The new in vivo experiments were conducted once due to two main constraints. First, the experimental timeline, including the time required to obtain CFU data, was approximately two months, which matched the time available for this revision. Second, we faced challenges in obtaining a sufficient number of animals from the conditional knockout strain, as these mice are poor breeders. Despite this, the *in vivo* data from the conditional knockout mice aligned with the *ex vivo* assays, where neutrophils isolated from these mice demonstrated that FAO blockade enhanced bacterial clearance. These two complementary approaches reinforce the conclusion that fatty acid metabolism impairs neutrophil control of Mtb. Hence, these findings ascertain the reproducibility of the results from two independent methods.

Reviewer's Comments: A recent study published in mucosal immunology supports some of the finding here (PMID: 38844208) - this study should at the very least be cited/mentioned/discussed as it provides additional support for the model proposed by the authors.

Response to reviewer: We have discussed this paper on Line 586 (Ref. 55, Andrews et al.) and have now added the following sentence to the Discussion: Line 586-596. This now reads as

"This aligns with recent findings showing that mitochondrial metabolism and lipid uptake pathways are upregulated in activated lung neutrophils, which overexpress CD36, enabling increased lipid uptake and creating a permissive niche for Mtb. Targeting these lipid uptake pathways has been shown to enhance the capacity of lung neutrophils to control Mtb replication[55]. Our study supports this observation and further demonstrates that inhibiting mitochondrial fatty acid metabolism can improve bacterial control by neutrophils and reduce TB pathology. Previous work from our group highlighted that neutrophil-rich TB lesions provide a nutrient-rich environment that supports bacterial replication by reducing oxidative and nitrosative stress, thus facilitating bacterial survival[8]. Our current study supports these findings, as evidenced by the predominance of replicative bacteria within these cells, highlighting the detrimental role of lipid metabolism of neutrophils in TB pathogenesis".

Part II – Major Issues: Key Experiments Required for Acceptance
Please use this section to detail the key new experiments or modifications of existing experiments that should be absolutely required to validate study conclusions.

Generally, there should be no more than 3 such required experiments or major modifications for a "Major Revision" recommendation. If more than 3 experiments are necessary to validate the study conclusions, then you are encouraged to recommend "Reject".

Reviewer #1

Reviewer's Comments: I do not see information regarding the SSB-GFP quantification (number of images and number of animals) added to the figure legends, even though that was stated as now included? (Needed information is noted as having been added to the methods.) There are many other instances where information is stated in the response to reviewers document as having been added to the manuscript, but are not actually present in the manuscript, with the referenced line numbers also correspondingly off. I am not sure if the wrong version of the revised manuscript was uploaded? All changes noted in the response to reviewer document should be carefully checked as indeed having been added as stated. In general, the figure legends also remain too sparse for effective understanding by readers without constant searching in the results/methods section for explanations. Just as one example, it is still not clear what the blot in Fig. 2G is showing. 2 different samples for -Mtb and 3 samples for +Mtb? From different experiments, or just repeat runs off the same experiment. Please edit throughout to improve ease of reader understanding.

Response to reviewer: We apologize for this lack of clarity in our manuscript. As recommended by the reviewer, we have mentioned the number of images/ mice used for confocal microscopy in all our Figure legends:

Fig. 1- Lines 1043-1045. Reads as
"For confocal microscopy images (C, F), representative image of n=3 mice / group; For (G) BL/6 n=3 mice/ group, for C3H n=4 mice/group; 3 fields of view per mouse; scale bar (C, G) 5µm, (F) 35µm; 200-500 bacterial rods per image were enumerated. Error bars depict Mean ± SD."

Fig. 4- Lines 1121-1124. Reads as
(E) Visualization and quantification of replicating bacteria (GFP+ on mCherry+ bacilli) in lung sections from infected untreated and inhibitor treated C3H mice at 29 dpi (Scale- 5µm). Representative image of n=4 mice / group, 3 fields of view per mouse, 200-500 bacterial rods per image, from two experiments.

Supplementary Fig. 2C- Line 1439-1440. Reads as
Sample sizes were **n=3 mice per group; 3 fields of view per mouse** for (C) and n=4 rabbits per group for (D). Error bars represent Mean ± SD.

Supplementary Fig. 7- Lines 1533-1539. Reads as
(A) Confocal microscopy images of lung sections (top to bottom) from control, etomoxir, ranolazine, mildronate, TMZ, and 2-DG treated mice (from **Fig. 4A**). Mtb is visualized using *smyc*'::mCherry, SSB foci are visualized by GFP, neutrophils are stained with Ly6G-Cy5, and nuclei are counterstained with DAPI.
(B) Total cell fluorescence of Ly6G staining was quantified by Image J.
Representative image of n=3 mice / group, 3 fields of view per mouse, 200-500 bacteria per image. Error bars indicate Mean ± SD; Scale- 5µm.

Fig. 2G- Western Blot, we have now added sample size in Lines 1069-1072. Now reads as

"Western Blot for Cpt1a (top) and Ubiquitin (bottom) from *ex-vivo* infected neutrophils at 4- and 24-hours pi. Each lane represents a biological replicate of a treatment condition, sourced from an independent well of a 12-well plate. sample size of n=2-3 replicates per group, representative of two independent experiments".

We have ensured that sample sizes and experiment repetitions are clearly stated in all figure legends. To avoid redundancy, this information is provided at the end of each figure legend, referencing the relevant data in the subpanels and specifying the sample sizes used to generate the data.

Reviewer's Comments: The anti-IL1R antibody treatment experiments are quite confusing as currently described, as while the neutrophil phenotypes track between +/- anti-IL1R treatment, other aspects do not. In Fig. S10B in particular, the Cpt1aΔneut mice have opposite phenotypes to their littermate controls depending on whether there is anti-IL1R treatment (i.e. in the absence of anti-IL1R treatment, there are higher numbers of infected monocytes, macrophages, eosinophils, and dendritic cells in the Cpt1aΔneut mice versus control, but the opposite is true in the presence of anti-IL1R treatment). How does that track with the decreased CFUs noted in Fig. 6B? At minimum, further explanation to put the results in better context is required for this set of experiments.

Response to reviewer: We thank the reviewer for bringing up this point. We have now revised the text to discuss the observed results, in Lines 432-446. This now reads as

"Cpt1a$^{\Delta neut}$ mice demonstrated a significantly lower bacterial burden, reduced neutrophil infiltration, and fewer Mtb-infected neutrophils compared to control littermates. In these mice, the bacterial niche appeared to shift toward other myeloid cells, likely due to the reduced numbers of total and Mtb-infected neutrophils, which serve as a major myeloid niche for *Mtb*, even in the BL/6 background. Interestingly, in the Cpt1a$^{\Delta neut}$ mice with IL-1R1 inhibition in the lungs, we observed a reduction in inflammatory pathology, along with a marginal but statistically significant reduction in bacterial load compared to control littermates (**Fig. 6A-B**). This decrease in lung pathology and bacterial burden was correlated with an overall reduction in the total number of myeloid cells, including Mtb-infected neutrophils (**Fig. 6C-F, Supplementary Fig. 10A, B**). While the reduction in other myeloid cells indicate a potential role of IL-1R signaling in regulating their infiltration, the reduction in neutrophils in the IL-1R inhibited lung was likely a result of Cpt1a inhibition, as this phenotype was observed in both the wild type and IL-1R1 inhibited lungs. Additionally, we observed an increase in the frequency of CD3+T-cells and CD19+B-cells in the lungs of untreated and IL1R-depleted Cpt1a$^{\Delta neut}$ mice, when compared to their respective control littermates following Mtb infection (**Supplementary Fig. 10C, D**). These findings underscore the detrimental role of Cpt1a-dependent mitochondrial fatty acid metabolism in neutrophils in modulating immune responses and controlling Mtb infection."

Reviewer #2: No major issues.
Reviewer #3: (No Response)

Part III – Minor Issues: Editorial and Data Presentation Modifications
Please use this section for editorial suggestions as well as relatively minor modifications of existing data that would enhance clarity.

Reviewer #1

Reviewer's Comments: Lines 272-273: Something appears to be missing in the front part of the sentence - "Following 4-hour pi to remove extracellular bacteria,…" What was done?

Response to reviewer: We are sorry for the lack of clarity in our writing. We rephrased this to make it clear. The revised text now reads

"To assess the impact of Mtb infection on neutrophils, naïve bone marrow-derived neutrophils were isolated and infected *ex-vivo* with *Mtb*. 4-hour post infection (hpi), non-phagocytosed extracellular bacteria were removed, and the infected cells in culture were analyzed after 24 hpi".

This has been mentioned in our Methods- Line 692-695.
Figure legends- Line 1081-1084.

Reviewer's Comments: Line 355: "…demonstrated pivotal role of metabolism…" Add "the" or "a" in between "demonstrated" and "pivotal".

- Line 481: Remove "in" from "…while in mature neutrophils could…".

- Line 508: "which then passively transported…" Missing "is" between "which" and "then".

- Line 535: Add citations for the macrophage work.

- Line 541: "Suggesting", not "suggest".

Response to reviewer: We apologize for our oversight and thank the reviewer for such a thorough examination of our manuscript. We have now made these changes in the new version of the manuscript.

Reviewer's Comments: Fig. S10A is not currently ever referenced in the text.
Response to reviewer: We apologize for not referencing this figure in our manuscript earlier. We have now referenced it in line 440 and have also discussed the results from **Supplementary Fig. 10A** there.

Reviewer #2: No minor issues.
Reviewer #3: (No Response)
